# Nucleotide-oligomerizing domain-1 (NOD1) receptor activation induces pro-inflammatory responses and autophagy in human alveolar macrophages

**DOI:** 10.1186/1471-2466-14-152

**Published:** 2014-09-25

**Authors:** Esmeralda Juárez, Claudia Carranza, Fernando Hernández-Sánchez, Elva Loyola, Dante Escobedo, Juan Carlos León-Contreras, Rogelio Hernández-Pando, Martha Torres, Eduardo Sada

**Affiliations:** Department of Microbiology, Instituto Nacional de Enfermedades Respiratorias Ismael Cosío Villegas, México City, México; Bronchoscopy Service, Instituto Nacional de Enfermedades Respiratorias Ismael Cosío Villegas, México City, México; Department of Experimental Pathology, Instituto Nacional de Ciencias Médicas y Nutrición Salvador Zubirán, México City, México

**Keywords:** Human alveolar macrophages, Innate immunity, NOD1, IRGM, LC3, Autophagy, *Mycobacterium tuberculosis*

## Abstract

**Background:**

Nucleotide-binding oligomerizing domain-1 (NOD1) is a cytoplasmic receptor involved in recognizing bacterial peptidoglycan fragments that localize to the cytosol. NOD1 activation triggers inflammation, antimicrobial mechanisms and autophagy in both epithelial cells and murine macrophages. NOD1 mediates intracellular pathogen clearance in the lungs of mice; however, little is known about NOD1’s role in human alveolar macrophages (AMs) or its involvement in *Mycobacterium tuberculosis* (Mtb) infection.

**Methods:**

AMs, monocytes (MNs), and monocyte-derived macrophages (MDMs) from healthy subjects were assayed for NOD1 expression. Cells were stimulated with the NOD1 ligand Tri-DAP and cytokine production and autophagy were assessed. Cells were infected with Mtb and treated with Tri-DAP post-infection. CFUs counting determined growth control, and autophagy protein recruitment to pathogen localization sites was analyzed by immunoelectron microscopy.

**Results:**

NOD1 was expressed in AMs, MDMs and to a lesser extent MNs. Tri-DAP stimulation induced NOD1 up-regulation and a significant production of IL1β, IL6, IL8, and TNFα in AMs and MDMs; however, the level of NOD1-dependent response in MNs was limited. Autophagy activity determined by expression of proteins Atg9, LC3, IRGM and p62 degradation was induced in a NOD1-dependent manner in AMs and MDMs but not in MNs. Infected AMs could be activated by stimulation with Tri-DAP to control the intracellular growth of Mtb. In addition, recruitment of NOD1 and the autophagy proteins IRGM and LC3 to the Mtb localization site was observed in infected AMs after treatment with Tri-DAP.

**Conclusions:**

NOD1 is involved in AM and MDM innate responses, which include proinflammatory cytokines and autophagy, with potential implications in the killing of Mtb in humans.

**Electronic supplementary material:**

The online version of this article (doi:10.1186/1471-2466-14-152) contains supplementary material, which is available to authorized users.

## Background

Pathogen recognition and induction of innate immune responses are important for the efficient elimination of infection. The nucleotide-oligomerizing domain 1 (NOD1) pattern recognition receptor senses the cytosolic presence of meso-diaminopimelic acid (DAP)-containing peptidoglycan fragments derived predominantly from the cell walls of gram-negative bacteria and *M. tuberculosis* [1, 2]. Following microbial sensing, NOD1 directly recruits a serine–threonine kinase, Receptor-interacting protein 2 (Rip2), which initiates a signal cascade that ultimately allows NF-κB to translocate to the nucleus [3]. Stimulating NOD1 induces the secretion of proinflammatory cytokines and chemokines (IL-6, IL-8, CXCL1, MIP-2, CCL2, and CCL5), the production of anti-microbial peptides (β-defensins), and autophagy in human epithelial cells [4–6].

Recent evidence reveals a major role for NOD1 in the resolution of respiratory infections. NOD1-deficient mice have an impaired ability to eliminate pulmonary *Legionella pneumophila* and to recruit neutrophils to the lungs [7]; clearance of *Streptococcus pneumoniae* and *Haemophilus influenzae* also occurs via a NOD1-dependent manner in a murine model of co-infection [8]. Rip2-/- mice show reduced iNOS expression and delayed neutrophil recruitment to the lungs and an inability to clear *Chlamydophila pneumoniae* infections, which subsequently lead to an increased rate of mortality [9]. Despite the evidence for NOD1’s role in resolving pulmonary infections, there are no data to support its role in *M. tuberculosis* infections.

Alveolar macrophages (AMs) are responsible for microbial lung clearance by orchestrating inflammatory responses that stimulate epithelial lung cells to produce additional chemokines and antimicrobial peptides, which amplify innate responses and help recruit other cells, such as monocytes and neutrophils [10]. AMs are also the cells responsible for eliminating *M. tuberculosis*. Although antimicrobial activity may be induced by NOD1, the involvement of AMs in NOD1-mediated responses and the full spectrum of cellular mechanisms responsible for antimicrobial NOD1-dependent activity, in mice and humans, remain to be elucidated.

In the present study, we investigate the presence of NOD1 in human AMs and examine its involvement with inflammatory cytokines and the induction of antimicrobial autophagy. For comparison, we include monocytes and monocyte-derived macrophages, as human monocytic cells have been reported to initiate proinflammatory responses following NOD1 ligand recognition [11]. Because NOD1 and autophagy are involved in intracellular pathogen clearance, we also analyze the role of NOD1 activation in the control of *M. tuberculosis* infection. Finally, we describe a novel role for NOD1 in primary human AM innate responses.

## Methods

### Ethics statement

The subjects for this study were healthy nonsmokers with a median age of 24 (range 20–40) years, 25% female, 75% male, seronegative for HIV-1, and no history of pulmonary or cardiac disease or recent infections. These subjects were studied after giving a signed informed consent, according to the Declaration of Helsinki, for bronchoalveolar lavage and venipuncture. The National Institute for Respiratory Diseases (INER) Institutional Review Board in Mexico City approved this protocol.

### Cells

Human bronchoalveolar cells were obtained by bronchoscopy, as previously described [12]. Cells were collected in sterile saline solutions and centrifuged at 400 × g for 15 minutes at 4°C. The pellets from the bronchoalveolar cells were suspended in culture medium, and the viability of the bronchoalveolar cells was assessed by Trypan blue exclusion (>98% in all cases). Bronchoalveolar cells were found to be 94.3 ± 2.8% alveolar macrophages according to flow cytometric analysis using a gate based on size and granularity. Therefore, we will refer to these cells as alveolar macrophages (AMs) in this study.

Monocyte-derived macrophages (MDMs) were obtained from peripheral blood mononuclear cells (PBMCs) that were prepared by centrifugation of whole heparinized venous blood diluted 1/1 with RPMI 1640 (Lonza, Walkersville, MD, USA) over a Lymphocyte separation solution (Lonza) gradient. PBMCs were plated in polystyrene dishes and incubated for 1 h at 37°C, in 5% CO_2_. After discarding non-adherent cells and extensive washings, the monocytes were recovered using a cell lifter. The viability of the monocytes was assessed by Trypan blue exclusion and was greater than 98% in all cases. MN concentrations were adjusted to 10^6^ cells/ml and were incubated on 24-well plates at 37°C, in 5% CO_2_ for 7 days, which allowed them to differentiate into MDMs that adhered to plastic [13]. MNs and MDMs were assayed for NOD1 activation. In all of the experiments, the culture medium consisted of RPMI 1640 supplemented with 50 μg/mL gentamycin sulfate, 200 mM L-glutamine, and 10% heat-inactivated pooled human serum.

### Cell stimulation

To assess ligand-induced responses, 10^6^ AMs, MDMs, and MNs were cultured in a final volume of 1 mL in an ultralow attachment polystyrene 24-well plate (Corning Inc., NY, USA). The cells were stimulated using 5 μg/mL of synthetic L-Ala-γ-D-Glu-meso-diaminopimelic acid (Tri-DAP) (InvivoGen, San Diego, CA, USA) for 24 h. Next, the supernatants were collected and kept frozen until the cytokine assessment; the cells were harvested and prepared for protein or mRNA extraction. Culture medium alone was used as a negative control, and LPS was used as a positive control, as indicated. In selected experiments, 10 μM of Rip2/p38 inhibitor SB203580 (Promega, Madison, WI, USA), 20 μM of PI3K inhibitor LY294002 (Promega) and 20 μM of pan-caspases inhibitor Z-VAD-fmk (Calbiochem, La Joya, CA, USA) were added to the cells 30 minutes before Tri-DAP stimulation to block NOD1 signaling.

### Reverse transcription and real-time PCR for gene expression

Total RNA was extracted and reverse transcribed as previously reported [13]. The cDNA was subjected to quantitative real-time PCR (qRT-PCR, TaqMan) to determine the NOD1, LC3 and IRGM mRNA expression levels using the comparative threshold cycle (ΔΔCt) as described previously [14]. Real-time PCR reactions were performed in duplicate wells according to the manufacturer’s protocol for Taqman predesigned gene assays; NOD1 (Hs01036717_m1), LC3 (Hs00171082_m1 and IRGM (Hs01013699_s1) were purchased from Applied Biosystems (Carlsbad, CA, USA). The Ct values for each gene were normalized to the endogenous control gene 18S rRNA (4319413E).

### Cytokine detection

Supernatants of 24-h cultures were assayed for the release of IL1β, IL6, IL8, IL10, IL12p70, IFNα2, and TNFα cytokines using the Milliplex human cytokine detection kit (Millipore, Billerica, MA, USA) according to the manufacturer’s protocol.

### Immunoblot

Proteins extracted from cytoplasmic lysates were separated by SDS-PAGE and transferred to polyvinylidene difluoride membranes, as previously described [14]. Briefly, membranes were blocked and incubated with the following antibodies: anti-human NOD1 (AdB serotec, Raleigh, NC, USA), IRGM (Abcam, Cambridge, MA), Atg9, and LC3 (Novus Biologicals, Littleton, CA, USA) or α-tubulin (Sigma-Aldrich, St. Louis, MO, USA) for 2 h followed by an incubation with peroxidase-conjugated anti-rabbit or anti-mouse IgG antibody (Sigma-Aldrich) for 1 h at room temperature. Specific bands were detected with the chemiluminescence SuperSignal system (Thermo, Rockford, IL, USA) and were revealed using autoradiographic films. Densitometry was performed using ImageJ 1.44o (National Institutes of Health, USA).

### Transduction of p62 to assess autophagic flux

MDMs were seeded in 8-well chamber slides (Thermo) at 5 × 10^5^ cells/well. The cells were transduced with 15 viral particles per cell for ectopical expression of p62-RFP (Premo Autophagy Sensor p62 kit, Molecular Probes, Carlsbad, CA). After 18 h, the cells were stimulated with 5 μg/ml of Tri-DAP and incubated for 5 or 24 h at 37°C and 5% CO_2_. Chloroquine 60 μM and medium were used as controls. We used Lysotracker (Molecular Probes) to stain lysosomes and Hoescht 33342 (Enzo Life Sciences, Farmingdale, NY) to stain nuclei following the manufacturer’s instructions. Cells were visualized in an AxioScope.A1 microscope (Carl Zeiss, Oberkochen, Germany) with the appropriate fluorescence filters. Images were acquired and analyzed with ZEN Pro software (Carl Zeiss).

### Infection with *Mycobacterium tuberculosis*and post-infection treatment

*M. tuberculosis* (Mtb) strain H37Rv (ATCC 25618) was grown as previously described [12]. AMs (10^5^) were infected with Mtb in RPMI with 30% non-heat-inactivated, pooled human AB serum at an infection ratio of 1–2 bacteria/20 cells in 96-well polystyrene plates; they were incubated for 1 h followed by three washes to remove any non-phagocytized bacteria. The cells were then cultured for another hour in RPMI supplemented with 10% heat-inactivated pooled human serum with or without 5 μg/ml of Tri-DAP. The infected macrophages were incubated after Tri-DAP treatment for 1 h (Day 0), 24 h (Day 1) and 96 h (Day 4) to evaluate the effects of macrophages on mycobacterial intracellular growth by quantifying the colony-forming units (CFUs). The intracellular growth index was calculated as the ratio of CFUs at Day 1 or 4 relative to the CFUs at Day 0.

AMs were infected with a multiplicity of infection (MOI) of 5 using 4 × 10^6^ cells cultured in polypropylene tubes under the same conditions described above and analyzed by transmission electron microscopy. Cells were treated with Tri-DAP with or without prior inhibition of Rip2/p38 and PI3K for 24 h post-infection and fixed in preparation for electron microscopy detection and subcellular localization of proteins.

### Electron microscopy

The subcellular localization of IRGM and LC3 proteins was performed using transmission electron microscopy (TEM), as previously described [15]. Briefly, cells were fixed in 4% paraformaldehyde in 0.2 M Sörensen buffer; the samples were dehydrated with increasing concentrations of ethylic alcohol and infiltrated with LR-White hydrosoluble resin (London Resin Co., Hampshire, United Kingdom). Sections that were 60- to 80-nm-thick were placed on nickel grids. The grids were incubated overnight at 4°C with specific polyclonal rabbit anti-IRGM (Abcam) and anti-LC3 (Novus Biologicals) antibodies followed by a 2 h incubation at room temperature with goat anti-rabbit IgG (Sigma-Aldrich) conjugated to 5-nm gold particles (Sigma-Aldrich) and diluted 1:20 in PBS. The grids were contrasted with uranyl acetate (Electron Microscopy Sciences, Fort Washington, PA) and examined with an M-10 Zeiss electron microscope (Karl Zeiss, Jena, Germany). To quantitatively assess autophagy protein recruitment to the mycobacteria-containing vesicle, we performed morphometric analyses by counting gold particles that colocalized with bacteria in 10 randomly selected cells from each condition (10–18 bacteria/condition) using ImageJ software.

### Statistical analysis

Data of paired samples from related subjects were analyzed by a non-parametric two-tailed Wilcoxon signed-rank test. Comparisons among non-related samples were analyzed using a Mann–Whitney U test. The colocalization of gold particles with bacteria between treatments was analyzed using a two-tailed paired *t*-test. The means and standard errors (SEs) were calculated where indicated. A p-value of p < 0.05 was considered a statistically significant difference. The statistical analyses were performed using SPSS 15.0 for Windows (SPSS, Chicago, IL, USA) and GraphPad Prism, version 5.0 (GraphPad Software Inc., San Diego, CA, USA).

## Results

### NOD1 is up-regulated in AMs and MDMs, but not in monocytes, after ligand stimulation

**Figure 1:**
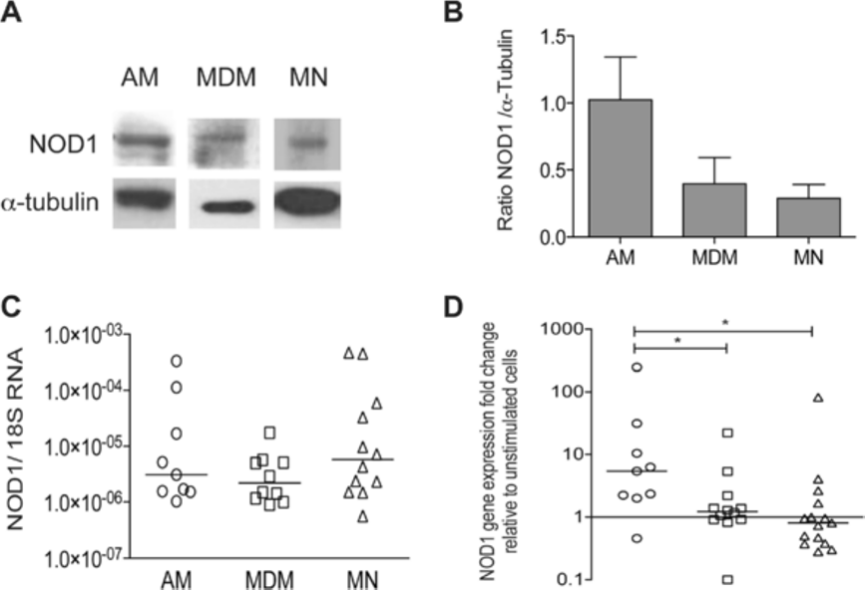
**Differential NOD1 expression and response patterns between differentiated macrophages and monocytes.** Unstimulated alveolar macrophages (AMs), monocyte-derived macrophages (MDMs) and monocytes (MNs) were lysed. NOD1 protein expression was measured from the cytosolic fractions by western blot analysis; one representative experiment out of three is depicted **(A)** and the mean ± SE of protein expression after normalized to tubulin is depicted, n = 3 **(B)**. Total RNA was extracted from cell lysates and reverse transcribed. NOD1 gene expression is depicted relative to the 18S RNA content of at least 9 subjects; the lines represent the median values **(C)**. Cells were incubated in the presence or absence of 5 μg/ml of Tri-DAP for 24 h. Up-regulation of NOD1 gene expression was assessed by quantitative PCR using the Taqman system and ΔΔCT for relative quantification. The fold changes of gene expression relative to the unstimulated cells of at least 9 subjects are depicted; the lines indicate the median values; *p < 0.05, Mann Whitney U test **(D)**.

Differences in Toll-like receptor patterns have been previously described between differentiated macrophages and monocytes [13]; therefore, we investigated whether NOD1 was expressed in unstimulated AMs, MDMs, and MNs. We observed a basal protein expression in all cell populations studied (Figure 1A) and densitometry revealed that NOD1 was more abundant in AMs (Figure 1B). We did not observe significant differences in the relative abundance of NOD1 mRNA in unstimulated cells (Figure 1C). However, after stimulation with Tri-DAP, AMs significantly up-regulated their NOD1 mRNA levels relative to the unstimulated cells, while MDMs barely modulated their gene expression and MNs showed down-regulated NOD1 levels (p < 0.05 vs. AMs, Figure 1D).

### NOD1 stimulation with Tri-DAP induces proinflammatory cytokines in AMs and MDMs

**Figure 2:**
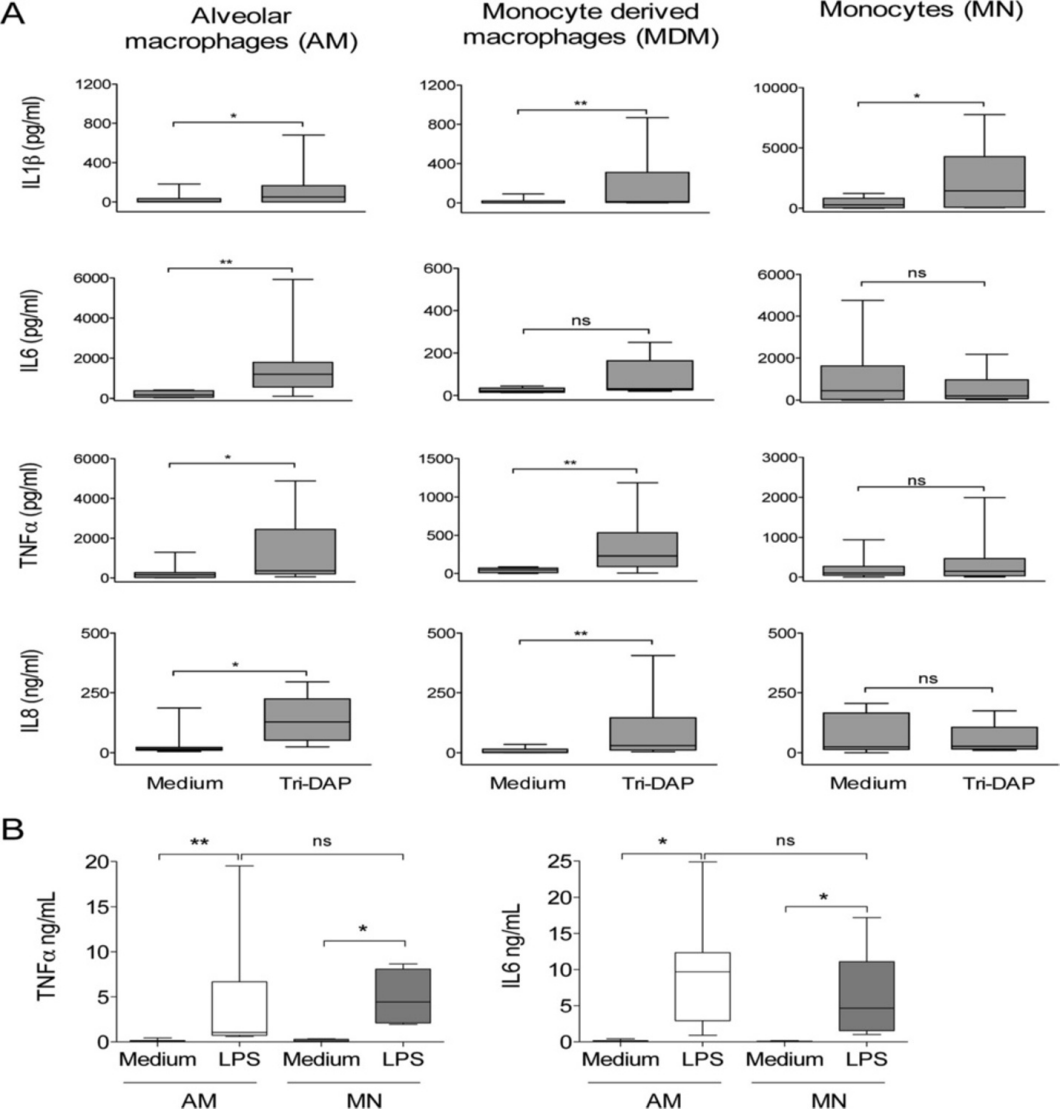
**Macrophages stimulated with Tri-DAP release proinflammatory cytokines.** Cells were incubated in the presence or absence of 5 μg/ml of Tri-DAP (n = 10, **A**) or 100 ng/ml of LPS (n = 8, **B**). The supernatants were collected after 24 h; cytokine production was measured using milliplex technology. Box plots indicate median and quartiles, *p < 0.05, **p < 0.01, ns = not significant Wilcoxon Rank test.

NOD1 pathogen recognition is typically accompanied by proinflammatory cytokine production [16]. AMs are responsible for initiating inflammatory responses, which recruit large numbers of neutrophils into the alveolar spaces to clear bacteria from entering terminal airways [17]. In this study, we have determined that Tri-DAP stimulation of macrophages in 24 h cultured supernatants results in pro-inflammatory cytokine production. We found that AMs release significant amounts of IL1β, IL6, TNFα, and IL8 (p < 0.05, Figure 2A). Meanwhile, MDMs released significant quantities of IL1β, TNFα, and IL8 (p < 0.05), and remarkably, MNs only produce significant amounts of IL1β in response to Tri-DAP stimulation (p < 0.05). Interestingly, in all of the cell types tested, we did not find any anti-inflammatory production of IL10 after Tri-DAP exposure (data not shown). In addition, AMs, the maximum responders to Tri-DAP stimulation, were also assayed for IL12p70, IFNα, and IL17; we found that AMs do not produce these cytokines in response to NOD1 ligand binding (data not shown). To confirm the selectivity of NOD1’s proinflammatory response, we stimulated AMs, the highest responders, and MNs, the lowest responders, with 100 ng/ml of LPS and observed that significant amounts of TNFα, IL6, and IL10 were released from both cell types (p < 0.05, compared to medium), and the levels of cytokine production were similar in both cells (Figure 2B). Cytokine responses were confirmed by qPCR using cell lysate-derived cDNAs (data not shown).

### Macrophages increase their autophagy activity after stimulation with NOD1 ligand

**Figure 3:**
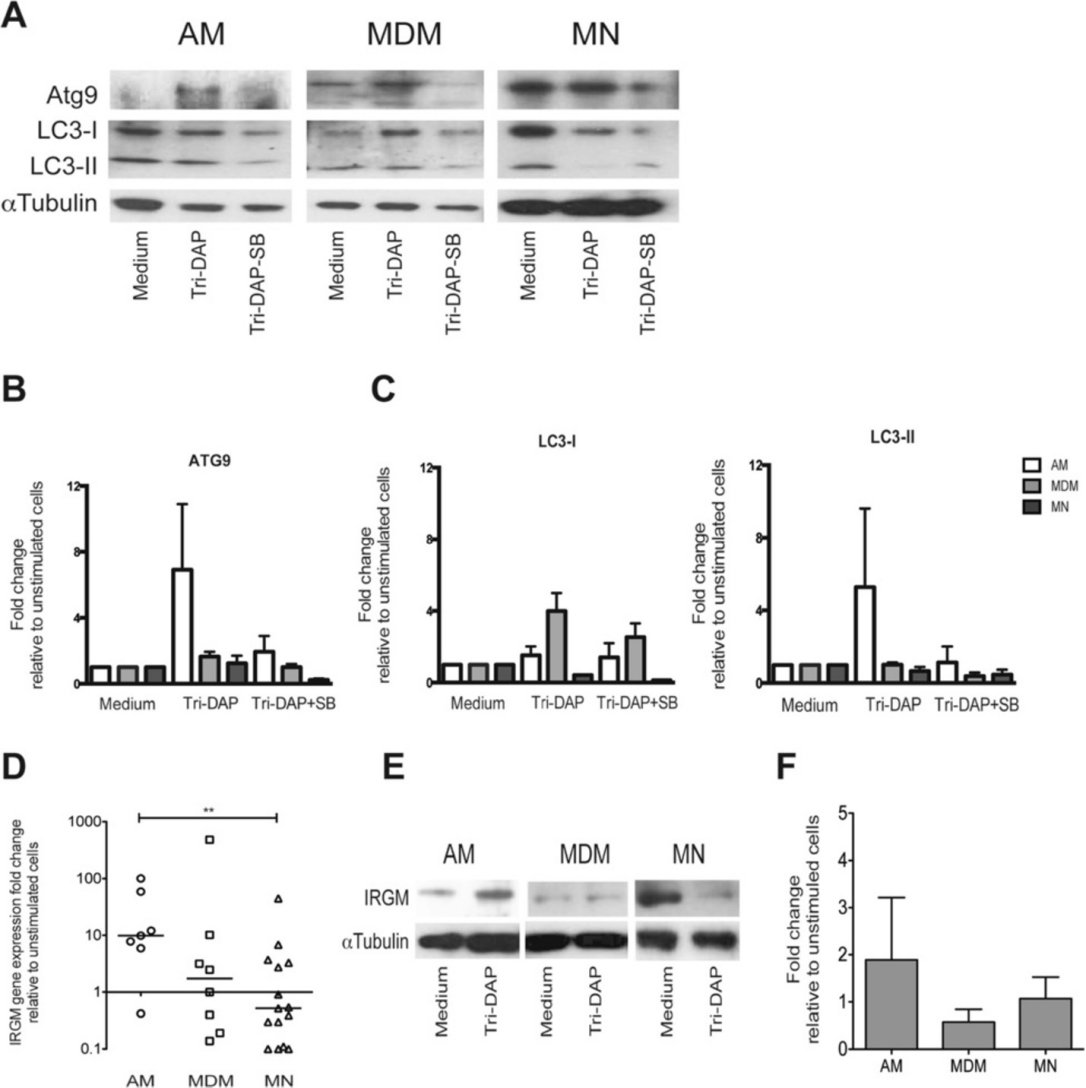
**After stimulation with the NOD1 ligand, macrophages increased their expression of autophagy proteins Atg9, LC3 and IRGM.** Alveolar macrophages (AMs), monocyte-derived macrophages (MDMs) and monocytes (MNs) were incubated in the presence of 5 μg/ml of Tri-DAP for 24 h. Cells were pre-incubated with Rip2/p38 inhibitor SB203580 (SB) for 30 min prior to Tri-DAP stimulation to block NOD1-mediated responses, as indicated. Atg9 and LC3 proteins were measured in the cytosolic fractions by western blot analysis **(A)**. The fold increases relative to the unstimulated cells were calculated after being normalized to tubulin, mean ± SE is depicted, n = 3 **(B, C)**. The up-regulation of IRGM gene expression was assessed after specific ligand recognition by quantitative PCR using the Taqman system and ΔΔCT method for relative quantification. The fold changes in gene expression relative to the unstimulated cells of at least 6 subjects are depicted; bold lines indicate medians, *p < 0.05 **(D)**. IRGM protein was measured in the cytosolic fractions of three subjects by western blot, and the fold increases relative to the unstimulated cells are indicated after being normalized to tubulin; mean ± SE is depicted, n = 3 **(E, F)**.

NOD1 induces autophagy in epithelial cells [6], and we recently found that NOD2 activation induces autophagy in human AMs as part of their antibacterial pulmonary defense [14]. Thus, we evaluated the ability of NOD1 to induce autophagy in different macrophages by determining the expression of the autophagy proteins Atg9 and LC3 after Tri-DAP exposure. We found that the expression of Atg9 increased after NOD1 stimulation in AMs and MDMs (Figure 3A). Pretreatment of AMs and MDMs with the Rip2/p38 inhibitor SB203580 for 30 min prior to Tri-DAP stimulation blocked NOD1-mediated responses and blocked the increased expression of Atg9 (Figure 3A,B). NOD1 activation also induced an increment in LC3-I and II in AMs and MDMs (Figure 3A,C). In contrast, no increase in autophagy proteins was observed in MNs. The enzyme IRGM is a key autophagy component with antimicrobial functions. Thus, we analyzed IRGM expression levels and determined that IRGM gene expression was elevated in AMs after Tri-DAP exposure, slightly increased in MDMs, and decreased in MNs (Figure 3D). Consistent with the gene expression data, only the AMs overexpress IRGM protein after Tri-DAP stimulation (Figure 3E,F).

**Figure 4:**
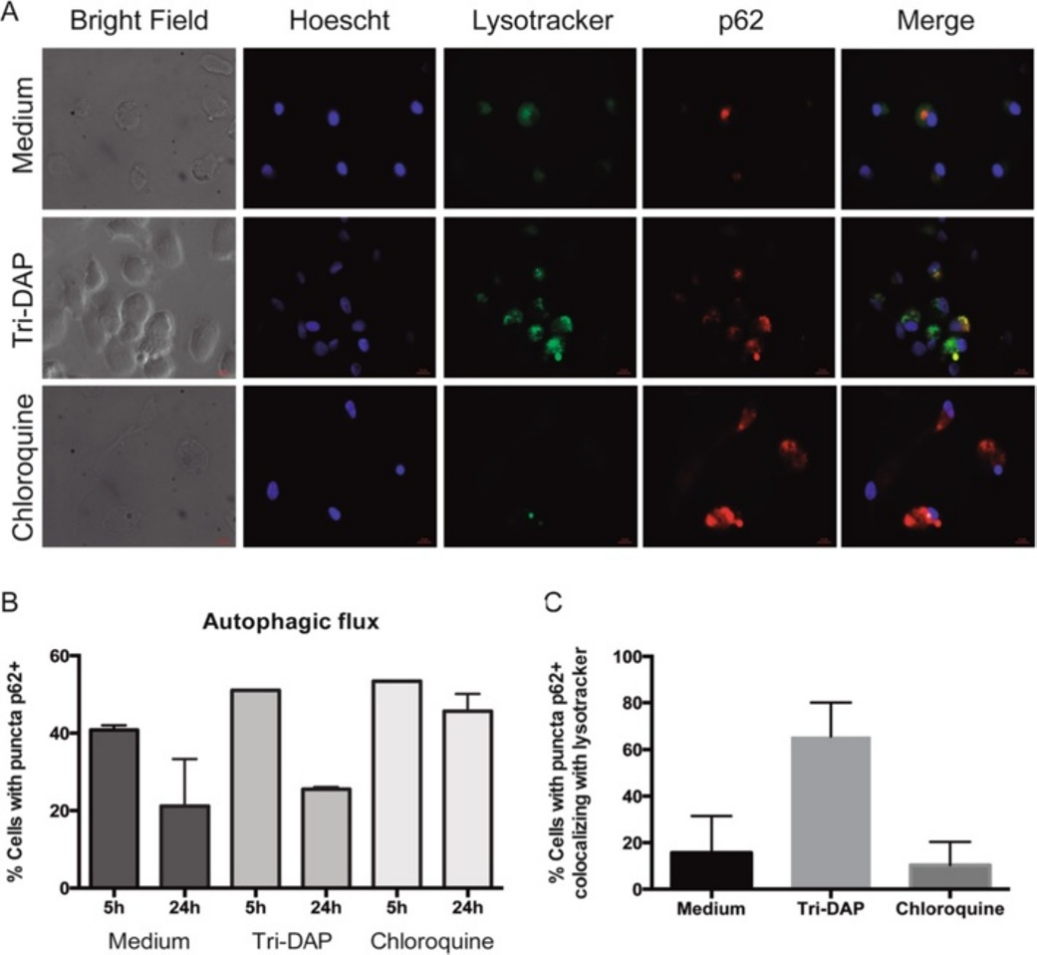
**Autophagy induced after stimulation with the NOD1 ligand in MDMs was productive.** MDMs were transduced with p62-RFP during 18 h. Cells were incubated in the presence of 5 μg/ml of Tri-DAP for 24 h. Accumulation of p62 in autophagosomes (puncta) was evaluated by fluorescence microscopy **(A)**. Lysotracker and Hoescht were used to stain lysosomes and nuclei, respectively. Choloroquine 60 μM was included as autophagic flux blocker. Transduction levels of p62 (5 h) and autophagy induction (24 h) were evaluated by counting cells with p62 puncta **(B)** and colocalization with lysotracker **(C)**. The mean ± SE of two independent experiments is depicted. Up to 300 cells were counted for each condition.

To confirm that autophagy was productive, we used ectopically expressed p62 in MDMs as an indicator of autophagic flux. The p62/SQSTM1 is a receptor for cargo destined to be degraded by autophagy [18]. Because the vector does not replicate within mammal cells, p62 is degraded when autophagy occurs and an accumulation of p62 positive vesicles indicate lack of autophagic flux. After 24 h of Tri-DAP treatment we observed cells with p62 puncta (Figure 4A). When we compared p62 puncta values obtained at 5 h to those of 24 h an evident reduction in p62 was observed confirming the development of productive autophagy (Figure 4B). In addition, most of the p62 puncta of Tri-DAP treated cells colocalyzed with lysotracker (Figure 4C). Chloroquine, as expected, induced autophagy but blocked the fusion autophagosomes-lysosome thus preventing the completion of the autophagic flux.

### Tri-DAP stimulation confines *M. tuberculosis*to autophagic vesicles and helps control intracellular growth

**Figure 5:**
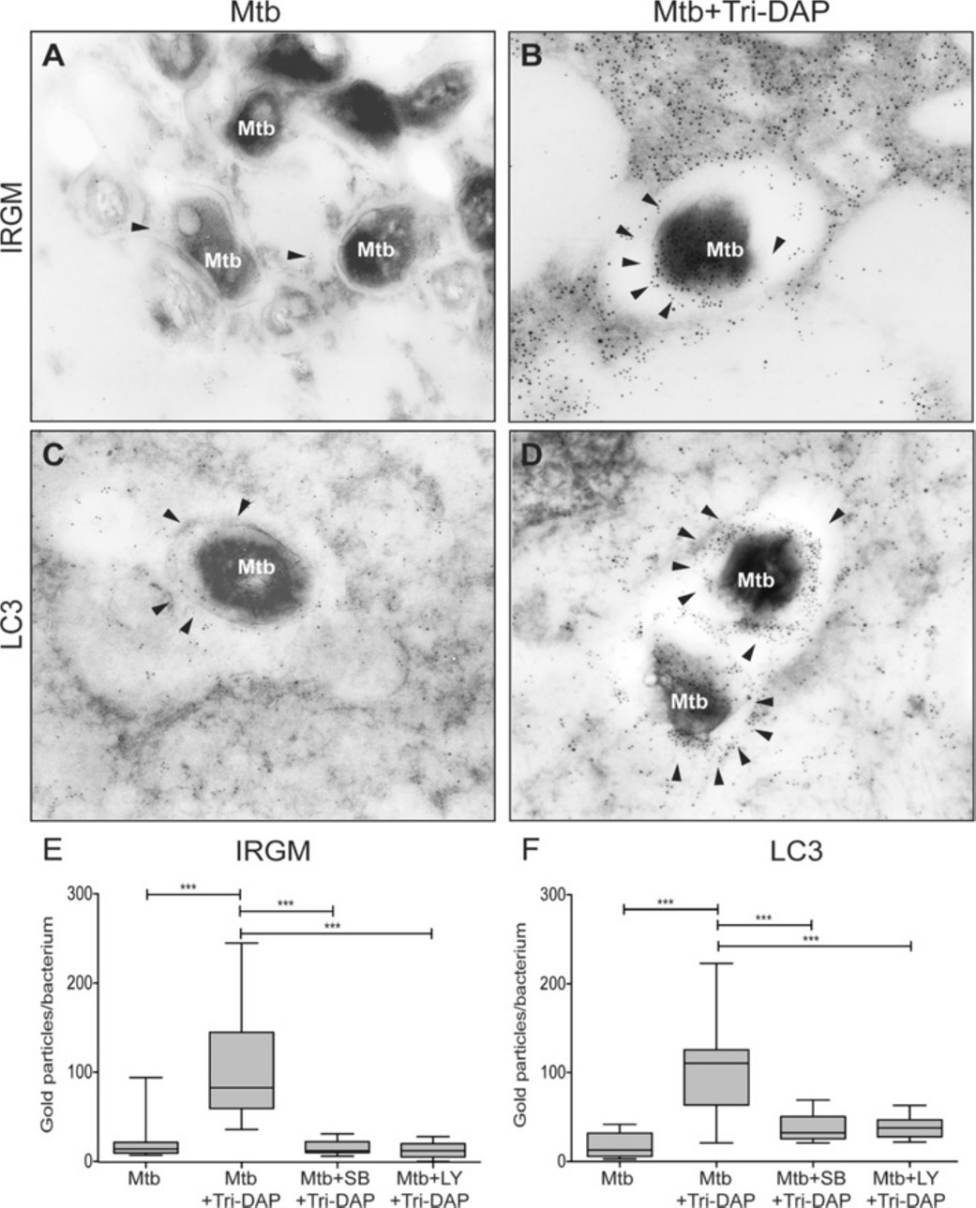
**NOD1-dependent induction of autophagy contributes to IRGM and LC3 recruitment to Mtb-containing vesicles.** AMs were infected with *M. tuberculosis* H37Rv using an infection ratio of 5 bacteria/macrophage for 1 h. Non-phagocytized bacteria were washed away. Then, the macrophages were treated with 5 μg/ml of Tri-DAP for 24 h. Cells were pre-incubated with Rip2/p38 inhibitor SB203580 (SB) or PI3K inhibitor LY294002 (LY) for 30 min prior to Tri-DAP stimulation which blocks NOD1- or autophagy-mediated responses, as indicated. The subcellular localization of autophagy proteins was observed in untreated **(A, C)** and Tri-DAP treated cells **(B, D)** using anti-IRGM and anti-LC3 antibodies, which were detected using a secondary antibody coupled to 5 nm gold particles (indicated with arrowheads; TEM X 62,000). Gold particles colocalizing with bacteria were manually counted in 10 macrophages of each condition **(E, F)** and the differences between the treatments are indicated: ***p < 0.01 and *p < 0.05 vs. Mtb, ++p < 0.01 vs. Tri-DAP, Wilcoxon Rank test. Box plots indicate median and quartiles.

Our results reveal that NOD1 activation induces an antibacterial state in AMs, which may improve the control of intracellular pathogenic infections. Therefore, we evaluated the role of NOD1 stimulation during *in vitro* infections with Mtb, one of the most successful intracellular pathogens. Previously, we demonstrated that Tri-DAP-treated AMs develop autophagy (Figures 3 and 4). Therefore, we used immunoelectron microscopy to examine the recruitment of IRGM and LC3 to Mtb-containing vesicles in AMs infected with Mtb that were treated or non-treated with Tri-DAP. Our results demonstrate that when AMs are treated with Tri-DAP post-infection, the Mtb is enclosed in autophagic vesicles that are positive for autophagy proteins, such as IRGM and LC3, whereas a minimum recruitment of these proteins is observed in the untreated macrophages (Figure 5A-D). To confirm whether the recruitment of IRGM and LC3, as well as autophagy, were dependent on NOD1 signaling, we pre-incubated cells with Rip2/p38 (SB) and PI3K (LY) inhibitors prior to Tri-DAP stimulation (Additional file 1). The use of both inhibitors significantly diminished the recruitment of autophagy proteins to the Mtb-containing vesicle (Figure 5E, F). In absence of stimulation the expression of IRGM was very low, and LC3 was homogeneously distributed within the cytosol (Additional file 2).

**Figure 6:**
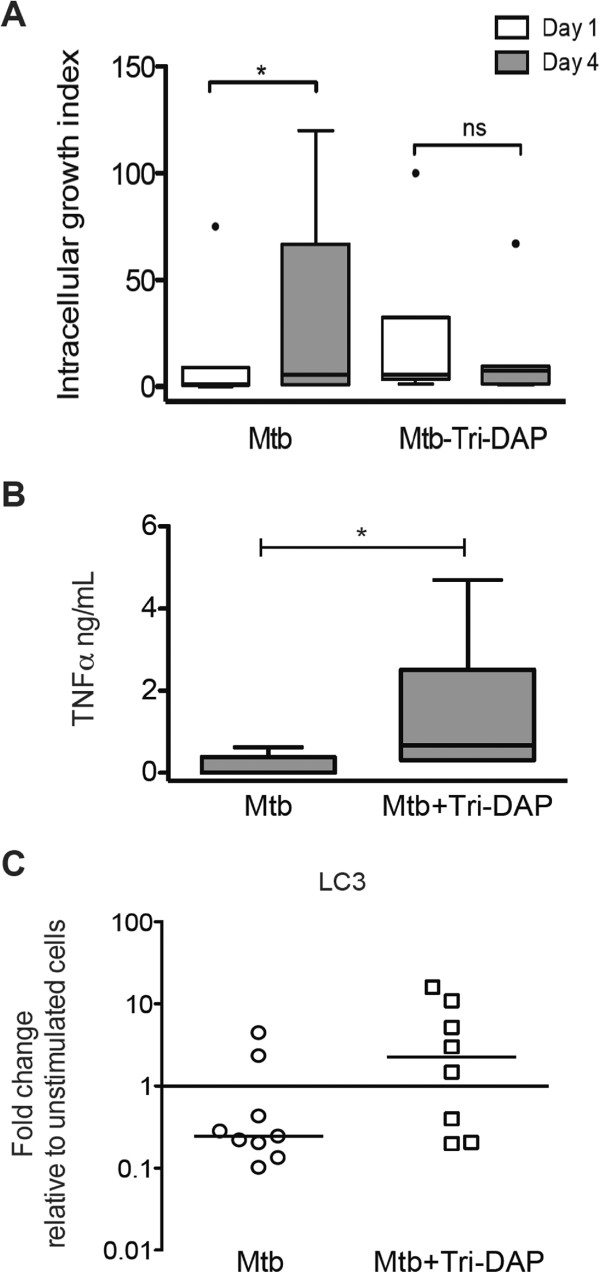
**Tri-DAP treatment contributes to intracellular growth control of mycobacteria in alveolar macrophages.** AMs were infected with *M. tuberculosis* H37Rv using an infection ratio of 1–2 bacteria/20 macrophages for 1 h. Non-phagocytized bacteria were washed away. Then, the macrophages were treated with 5 μg/ml of Tri-DAP for 24 h. The intracellular bacterial burden was measured by quantifying the colony-forming units, and the intracellular growth index was calculated after 1 and 4 days post-infection relative to the phagocytized bacteria at day 0; n = 7, *p < 0.05 **(A)**. TNFα production was determined by ELISA in 24 h supernatants, n = 7, *p < 0.05, box plots indicate median and quartiles **(B)**. LC3 gene expression was measured by quantitative PCR using the Taqman system and ΔΔCT method for relative quantification. The fold changes in gene expression relative to the unstimulated cells of at least 8 subjects are depicted; bold lines indicate medians **(C)**.

Next, we investigated whether Tri-DAP treatment improves the control of Mtb-intracellular growth. The intracellular growth index revealed that bacterial growth was lower inside AMs when they were treated with Tri-DAP post-infection (Figure 6A) compared with the infected AMs that did not receive Tri-DAP stimulation. Moreover, Tri-DAP stimulation induced the release of significant amounts of TNFα and the overexpression of LC3 mRNA in AMs infected with Mtb (Figure 6B,C) that were not observed in AMs when they were not treated with Tri-DAP.

## Discussion

The nucleotide-oligomerizing domain 1 (NOD1) pattern recognition receptor is essential in respiratory innate defense. NOD1 deficiencies cause severe respiratory diseases and impair antibacterial mechanisms in mouse models [7, 9]. Human lung epithelial cells contribute to the clearance of attenuated *K. pneumoniae* by producing beta-defensins in a TLR2- and NOD1-dependent manner, which highlights the importance of NOD1 in respiratory pathogen elimination and in pathogen-evasion mechanisms in human pneumonia [19]. However, the role of NOD1 in human AM responses remains unknown. In the current study, we investigate the role of NOD1 in the primary human AM innate response, focusing on the proinflammatory effectors and the induction of autophagy.

First, we determined the intracellular expression of NOD1 in unstimulated primary human AMs and compared their NOD1 expression levels with those of MNs and MDMs. We determined that NOD1 was expressed in the cytosol of AMs, MNs, and MDMs. The NOD1 mRNA levels in unstimulated AMs are similar to those in MDMs and MNs, indicating that NOD1 expression is independent of cell differentiation and that AMs, MNs, and MDMs could potentially respond to stimulation by NOD1 ligands. However, NOD1 gene expression was only up-regulated in AMs after Tri-DAP stimulation; neither the MDMs nor the MNs increased their NOD1 gene expression, which suggests that regulation or the functional ability of the receptor is cell type-dependent.

In agreement with ligand-induced NOD1 overexpression, we observed a broader proinflammatory cytokine profile triggered by NOD1 in AMs. Alveolar macrophages produce significant quantities of TNFα, IL6, IL1β, and IL8; MDMs produce all of these cytokines except IL6; however, the MN response is restricted to IL1β. The low cytokine response observed in MNs is consistent with previous reports, which indicate that human PBMCs or purified MNs do not release pro-inflammatory cytokines after low concentrations of Tri-DAP stimulation. Instead, they synergize with TLR ligands to induce strong cytokine responses. However, increased Tri-DAP concentrations induce cytokine responses in MNs [20, 21]. Thus, the cytokine responses elicited by NOD1 stimulation depend on the stage of maturation and the specific requirements of the tissue. AMs produce increased levels of IL8, which suggests a role for NOD1-dependent neutrophil recruitment to human lungs. Meanwhile, other reports suggest that NOD1 responses induce not only neutrophil recruitment but also an increased neutrophil-killing capacity [22, 23]. NOD1 induces the production of proinflammatory cytokines, which is critical for bacterial clearance in mice with *L. pneumophila* [24].

One of the antimicrobial mechanisms induced by innate receptors is autophagy, which is inducible by NOD1 in epithelial cells [6]. Therefore, we investigated whether macrophages could initiate autophagy in response to NOD1 ligand stimulation. We investigated the expression of the autophagy-related proteins Atg9, LC3, and IRGM after stimulation with Tri-DAP and found that AMs increase the expression of Atg9 and LC3. These results indicate that the autophagy process is involved because Atg9 is necessary for initiating autophagosome formation and LC3 is required to finalize autophagosome maturation [25, 26]. Moreover, the degradation of p62 confirms the autophagy completion. In addition, NOD1 induces the overexpression of IRGM in AMs, which implies an antimicrobial component because human and murine IRGM not only induce autophagy but also collaborate to eliminate intracellular pathogens, including Mtb [27, 28].

Taken together, our results suggest that AMs are highly responsive to NOD1 stimulation, MDMs elicit moderate innate responses after Tri-DAP stimulation, and MNs exhibit a limited response. Thus, although basal expression is similar, regulation of NOD1 expression levels, the quality and magnitude of NOD1-driven cytokine and autophagy responses are associated with the macrophage differentiation status and the tissue environment of the cell, which explains why higher responses are observed in AMs and MDMs.

Autophagy constitutes an important mechanism of defense against Mtb [29]. In this study, because autophagy was mainly induced in AMs, we evaluated the antimicrobial activity associated with autophagy in AMs. We infected AMs with Mtb as a model intracellular pathogen and evaluated the effect of NOD1 activation as an inducer of autophagy after an established infection. Some Mtb virulence factors inhibit autophagy in host macrophages to grant survival [30, 31]. Therefore, Tri-DAP was added post-infection to overcome Mtb-associated inhibition of autophagy. After treating infected AMs with Tri-DAP, we observed recruitment of autophagy indicators, such as IRGM and LC3, to the pathogen-containing vesicles in a Rip2-dependent manner. Rip2-dependent and -independent responses have been documented for NOD1 and NOD2 [6]. LC3 and IRGM up-regulation have been used to measure autophagy because they are induced in human AMs after NOD2 activation [14]. Autophagy proteins did not increase and they were not recruited to pathogen-containing vesicles in the cells incubated with Rip2/p38 inhibitor prior to Tri-DAP stimulation. Therefore, our results indicate that the NOD1 ligand also induces autophagy in a Rip2-dependent manner in AMs. Autophagosomes become degradation compartments that influence phagosome maturation and pathogen degradation [32]. Therefore, the NOD1-dependent formation of autophagosomes may have improved the control of the intracellular mycobacterial burden that we observed in AMs.

## Conclusions

The results of our study demonstrate the presence of NOD1 in AMs for the first time and show that this receptor is functionally active. These studies provide evidence that NOD1 induces the production of proinflammatory mediators and triggers autophagy mechanism in human alveolar macrophages. The antimicrobial profile activated by NOD1 may be of relevance, especially early after pulmonary microbe invasion. The induction of autophagy in AMs after NOD1 activation is a significant mechanism for eliminating intracellular microbes that enter the alveolar space, such as *M. tuberculosis*. NOD1 and NOD2 induce autophagy and recruitment of neutrophils and macrophages and have a role in defense against *M. tuberculosis* [33, 34]; therefore, ligands of both receptors are potential candidates for use as immunomodulators in tuberculosis infection. Further studies are needed to elucidate whether activating the NOD1 receptor also induces other aspects of the AM immune response.

## Authors’ information

Martha Torres and Eduardo Sada are senior authors.

## Electronic supplementary material

Additional file 1: **Tri-DAP-induced recruitment of IRGM (A-D) and LC3 (E-H) depends on NOD1 signaling and autophagy initiation.** (TIFF 18 MB)

Additional file 2: **Detection of basal levels of IRGM and LC3 in unstimulated uninfected cells.** (TIFF 14 MB)

## Abbreviations

AMs: Alveolar macrophages
MDMs: Monocyte-derived macrophages
MNs: monocytes
NOD1: Nucleotide-oligomerizing domain-1
IRGM: Immunity related GTPase M
Rip2: Receptor-interacting protein 2
p38: p38 mitogen-activated protein kinase
Tri-DAP: L-Ala-γ-D-Glu-meso-Diaminopimelic acid
LPS: Lipopolysaccharide.
