# Supplementary figures and images for: Nucleotide-oligomerizing domain-1 (NOD1) receptor activation induces pro-inflammatory responses and autophagy in human alveolar macrophages
Source: BMC Pulm Med. 2014 Sep 25;14:152. doi: 10.1186/1471-2466-14-152 (PMC4190423; doi:10.1186/1471-2466-14-152)

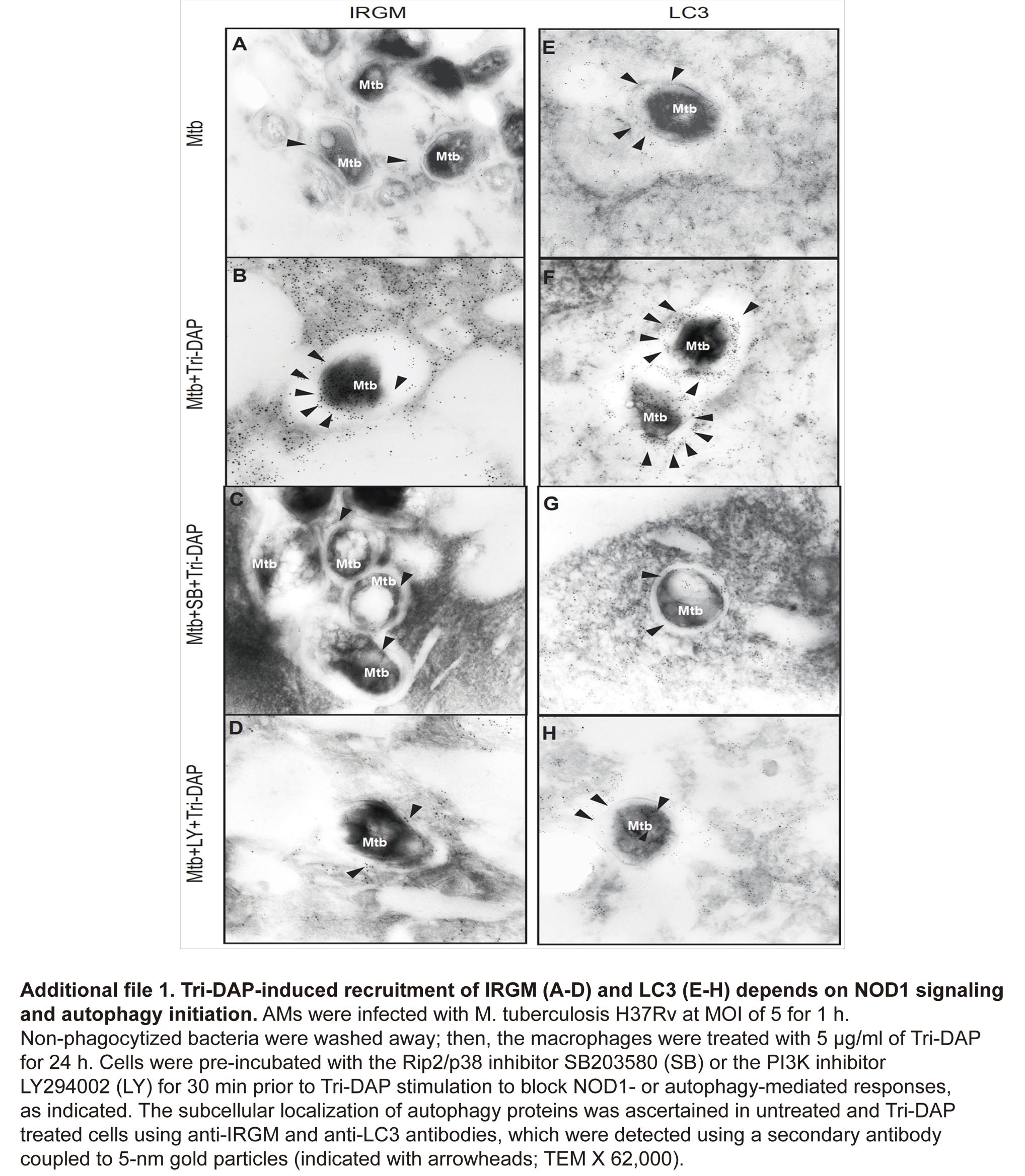

Supplement: Supplementary file 1 — Additional file 1: Tri-DAP-induced recruitment of IRGM (A-D) and LC3 (E-H) depends on NOD1 signaling and autophagy initiation. (TIFF 18 MB) [file 12890_2013_590_MOESM1_ESM.tiff]

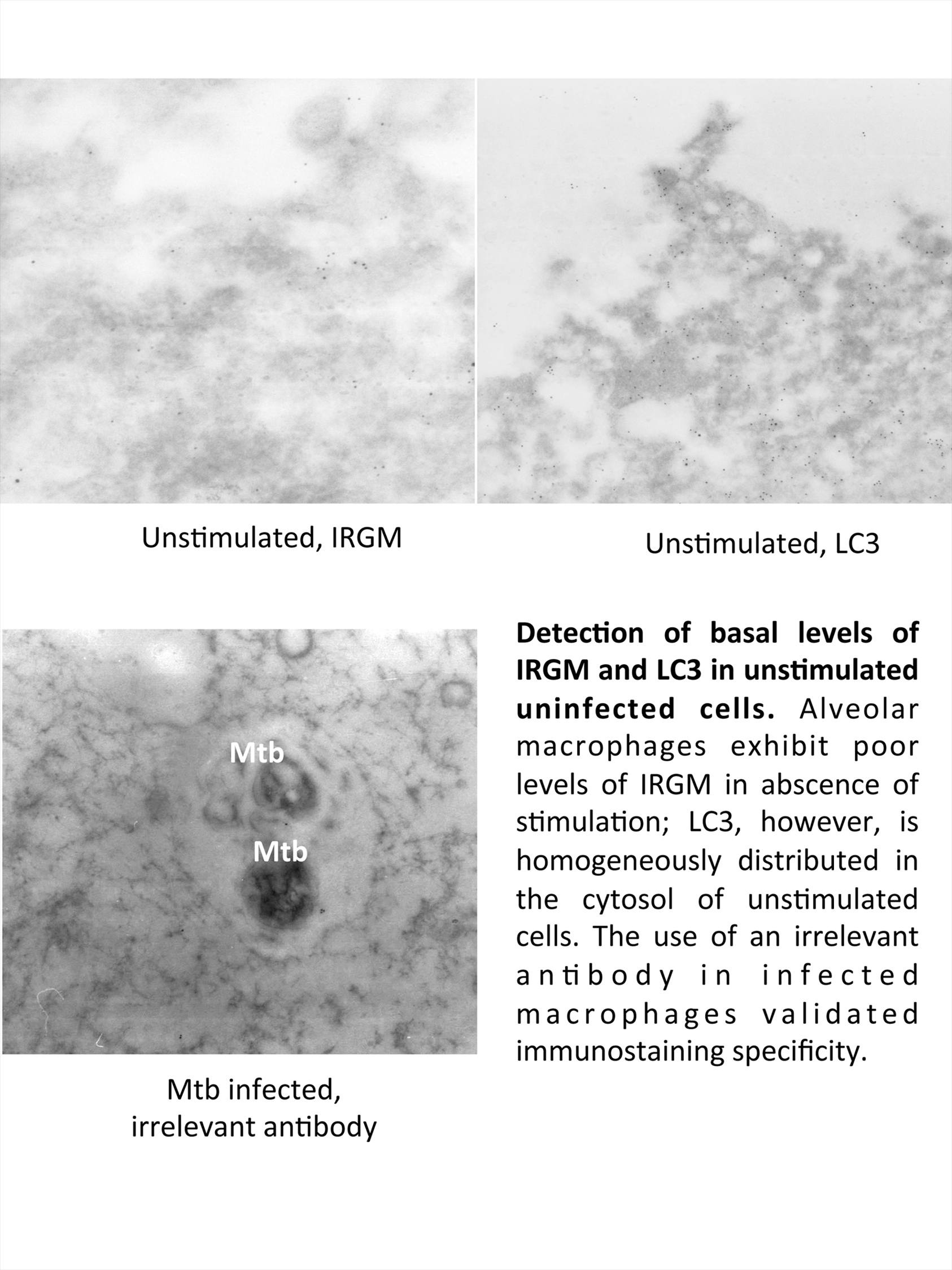

Supplement: Supplementary file 2 — Additional file 2: Detection of basal levels of IRGM and LC3 in unstimulated uninfected cells. (TIFF 14 MB) [file 12890_2013_590_MOESM2_ESM.tiff]
